# Sinoatrial Node Dynamics: Clinical Relevance and microRNA-Based Modulation of Pacemaker Activity

**DOI:** 10.7759/cureus.97101

**Published:** 2025-11-17

**Authors:** Hanan Nur

**Affiliations:** 1 Medicine and Surgery, Manchester University Foundation Trust, Manchester, GBR

**Keywords:** cardiology research, genetics, genetics and molecular biology, internal medicine-cardiology, medical education, medical school education

## Abstract

The sinoatrial node (SAN) functions as the primary pacemaker of the heart, initiating rhythmic electrical impulses that govern cardiac contraction. It consists of specialised pacemaker cells located between the superior vena cava and the right atrium, supported by a paranodal region that contributes to rhythm stabilisation and may serve as a secondary pacemaker during dysfunction. This review describes the structural organisation and molecular regulation of the SAN, with particular focus on the role of HCN4 ion channels and the influence of the microRNA miR-486-3p in modulating pacemaker activity. Understanding this relationship may support the development of novel therapeutic strategies for managing conditions such as sinus tachycardia, offering potential alternatives to existing pharmacological approaches.

## Introduction and background

The origin of the heartbeat was once a matter of extreme conjecture, a matter that has since been settled following the discovery of the sinoatrial node (SAN) [1-4]. In the normal human heart, the SAN functions as the primary pacemaker, and it has long been established that the SAN is typically located in a distinct region between the superior vena cava and right atrial junction [5,6]. While this is an appropriate description, it is only recently, however, that we have gained a real insight into the sheer complex anatomical and physiological nature of the SAN. Given its fundamental role in maintaining rhythmic cardiac activity, dysfunction of the SAN can lead to clinically significant arrhythmias such as sinus node dysfunction and inappropriate sinus tachycardia [6], underscoring the importance of understanding its structure and regulation.

In addition to detailing the anatomy of the SAN, this review aims to explain some of the key mechanisms governing the spontaneous impulse generation exhibited by the SAN and how its automaticity is regulated. Two interacting mechanisms govern the SAN’s pacemaking activity, often referred to as "clocks": the “calcium clock”, driven by intracellular calcium release, and the “membrane clock”, which plays a central role in initiating diastolic depolarisation [7]. Given its significance in early pacemaker activity, this review will focus primarily on the “membrane clock”. Furthermore, this review presents current insights into SAN regulation, with a particular emphasis on the emerging role of microRNAs in intrinsic SAN control and their therapeutic potential in managing sinus tachycardia, potentially addressing some limitations of existing treatments such as Ivabradine.

To produce a comprehensive review of the literature relating to SAN dynamics, its historical and clinical relevance, and microRNA-based modulation of pacemaker activity, a structured search was conducted using the PubMed database. Relevant keywords such as “sinoatrial node,” “pacemaker activity,” “paranodal region,” and “miR-486-3p” were used, and results were organised by publication date, covering studies from the earliest descriptions and discovery of the SAN through to the most recent insights into SAN structure, function, and microRNA regulation of pacemaker activity.

## Review

Discovery of the primary pacemaker of the heart

The ground-breaking work of Sunao Tawara, who mapped the connections within the cardiac conduction system [2], laid the foundation for the discovery of the SAN by Sir Arthur Keith and Martin Flack, a medical student at the time, in 1907 [3]. They identified a distinct structure in the right atrial auricle, which they initially termed the “sino-auricular node”. Further anatomical studies confirmed the presence of this structure in other mammalian hearts as well [3]. However, it was not until four years later that the SAN was definitively recognised as the site of cardiac impulse initiation and established as the heart’s primary pacemaker [4].

Anatomy of the human SAN

Anatomical Location of the SAN

**Figure 1:**
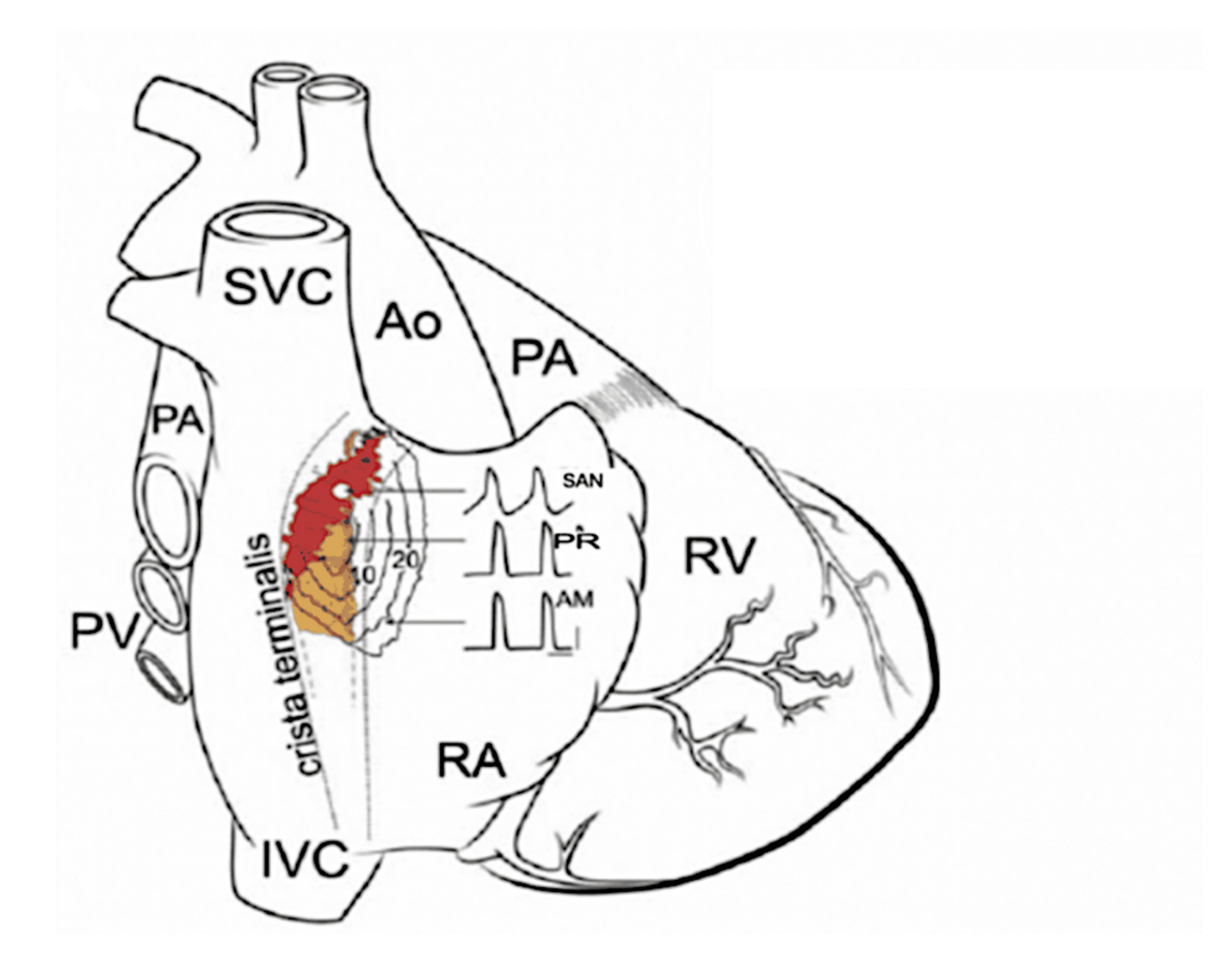
Model of a human heart illustrating the SAN position in the right atrium The red region demarcates the sinoatrial node (SAN) centre, the yellow area shows the paranodal area, and the white point denotes the primary pacemaker location. Action potentials from the atrial muscle (AM), paranodal region (PR), and sinoatrial node (SAN) are presented. SVC: superior vena cava, Ao: aorta, PA: pulmonary artery, PV: pulmonary veins, RV: right ventricle, RA: right atrium, IVC: inferior vena cava. Reproduced with permission from ref [11].

The anatomical position of the SAN is well characterised and consistently described in the literature. It is typically located in close proximity to critical anatomical landmarks, including the sinoatrial nodal artery and the right phrenic nerve [5]. Morphologically, the SAN presents as a crescent-shaped structure composed of specialised pacemaker tissue, situated at the junction between the superior vena cava and the right atrium, with its distal extent adjacent to the crista terminalis [6] (Figure 1). The proximal region and head of the SAN are typically confined to the subepicardial layer within the sulcus terminalis, embedded beneath epicardial adipose tissue. In contrast, the central and distal (tail) regions of the node penetrate obliquely through the crista terminalis, a prominent muscular ridge of the right atrium, thereby occupying a position closer to the endocardium [7]. This spatial configuration reflects the SAN’s complex three-dimensional architecture and its integration within both the epicardial and endocardial surfaces of the atrial wall.

Owing to its anatomical location, the SAN receives its arterial supply from the SAN artery, a vessel known for its considerable interindividual variability [8]. In approximately 55-60% of the population, the SAN artery originates from the right coronary artery (RCA), while in the remaining 40-45%, it arises as a branch of the left circumflex artery (LCx) [9]. As such, the course of the SAN artery as it approaches and enters the node can differ significantly among individuals [9]. Importantly, the SAN is typically positioned centrally along the trajectory of this artery [10]. Although the course of the SAN artery may vary in a structurally altered right atrium, it remains a key feature in identifying the SAN and has important surgical relevance. Accurate recognition of its path can help reduce the risk of iatrogenic injury to the node during interventional or surgical procedures [10].

Gross Structure of the SAN

As the sulcus terminalis, the groove in which a considerable portion of the SAN is located, is permeated predominantly with epicardial fatty tissue, the gross structure of the node is therefore not directly visible [11]. Through histological examination, three-dimensional tissue reconstruction, and SAN mapping, the shape of the human SAN has been determined to have a crescent-like appearance (Figure 1), revealing the compartmental structure of the SAN complex, consisting of a head, central body, and tail (most distal region), as well as a number of specialised electrical conduction pathways [7,12]. Sánchez-Quintana et al. (2005) described the absence of SAN insulation within a fibrous sheath and an SAN border that appears asymmetrical, consisting of numerous extensions interdigitating with transitional cells, an intermediate group of cells that provide a connection between the pacemaker cells and regular atrial myocardium [12]. Moreover, in this study, the SAN was reported to show variation in its mean length, measuring approximately 13.5 mm but ranging anywhere between 8 and 21.5 mm, with a width of around 2-6 mm in the average adult human heart [12]. However, the mean length of the SAN indicated by Mayatsuma et al. (2004) in their case subjects was longer and was described to measure approximately 21 mm [13]. Additionally, it has also been reported that there appears to be no relationship between the size of the SAN, right atrium size, and weight of the human heart [12]. Nevertheless, there is general consensus regarding gross physiognomies such as the size, crescent or banana-shaped three-dimensional structure of the SAN, and the distinct SAN artery [8,10-12,14].

Cells of the human heart 

General Organisation of Non-pacemaker Cells

In non-pacemaker regions of the heart, the predominant cellular components include cardiomyocytes, smooth muscle cells, endothelial cells, and cardiac fibroblasts [15]. Cardiac fibroblasts, derived from mesenchymal origins, contribute to the formation of the extracellular matrix, which provides essential structural support to the myocardium [16,17]. Smooth muscle cells maintain the integrity of vascular structures, while endothelial cells form the inner lining of the cardiac chambers, valves, and blood vessels [16]. Historically, smooth muscle and endothelial cells were considered to comprise a smaller proportion of the total cardiac cell population. However, recent studies challenge this view, suggesting that cardiac fibroblasts are less abundant than previously believed and that endothelial cells represent the most prevalent non-cardiomyocyte cell type in the human heart [18].

Despite variability in reported cell proportions, there is general agreement that cardiomyocytes account for approximately 25-35% of the total number of cardiac cells [19]. Notably, cardiomyocytes represent over 70% of the heart's total mass, owing to their substantially larger size compared to other cardiac cell types [19].

Structurally, cardiomyocytes differ markedly from SAN pacemaker cells. They are striated due to the presence of organised sarcomeres composed of overlapping actin and myosin filaments, which enable their contractile function, a feature absent in SAN cells [19]. Typically, cardiomyocytes exhibit a branched morphology with one or two centrally located nuclei and a high density of mitochondria to support their energy demands [20]. At the interfaces between adjacent cardiomyocytes are intercalated discs, which contain specialised gap junctions. These junctions facilitate ionic exchange, allowing for the rapid propagation of action potentials and coordinated depolarisation of the myocardium [21].

Cellular Architecture of the Human SAN

The SAN pacemaking cells are dispersed within a medium of connective tissue, primarily consisting of elastin and collagen tissue sheaths, and are found among fibroblasts [22]. The predominantly fibrous matrix in which the pacemaker cells are located causes the SAN to have an irregular border, facilitating its distinction from the adjacent non-pacemaker tissue of the right atrium [22]. In comparison, histological analysis shows that the right atrial tissue contains a considerably smaller aggregate of connective tissue [22]. In the average adult SAN, the connective tissue matrix contributes to 40-55% of the SAN architecture, and in addition to housing the pacemaker cells of the SAN, it also facilitates the mechanical shielding of the node [23]. Moreover, excluding the distinct conduction pathways that form electrical links between the pacemaker cells and the surrounding atria, a structural perimeter is formed around the SAN by layers of fatty tissue, fibrosis, or irregular myofibres (which may also be present alongside the other border tissue) [23]. This structural margin provides electrical insulation and shelters the pacemaker cells from the hyperpolarising force of the adjacent atrial myocytes [23]. Thus, it forms a conduction barrier that allows for the efficient maintenance and regulation of a regular sinus rhythm of 60-100 beats per minute (bpm) [23]. Furthermore, the centre of the human SAN contains a distinct collection of “P” cells, also referred to as the characteristic node cells, and these are understood to be the principal pacemaker cells of the SAN [24] (Figure 1).

The cells of the SAN are comparatively smaller in size and paler in contrast to the neighbouring atrial myocytes and are termed “empty cells” as they contain fewer sarcosomes, organelles, and sarcomeric apparatus, contributing to the lighter appearance of the node upon histological analysis [24,25].

**Figure 2:**
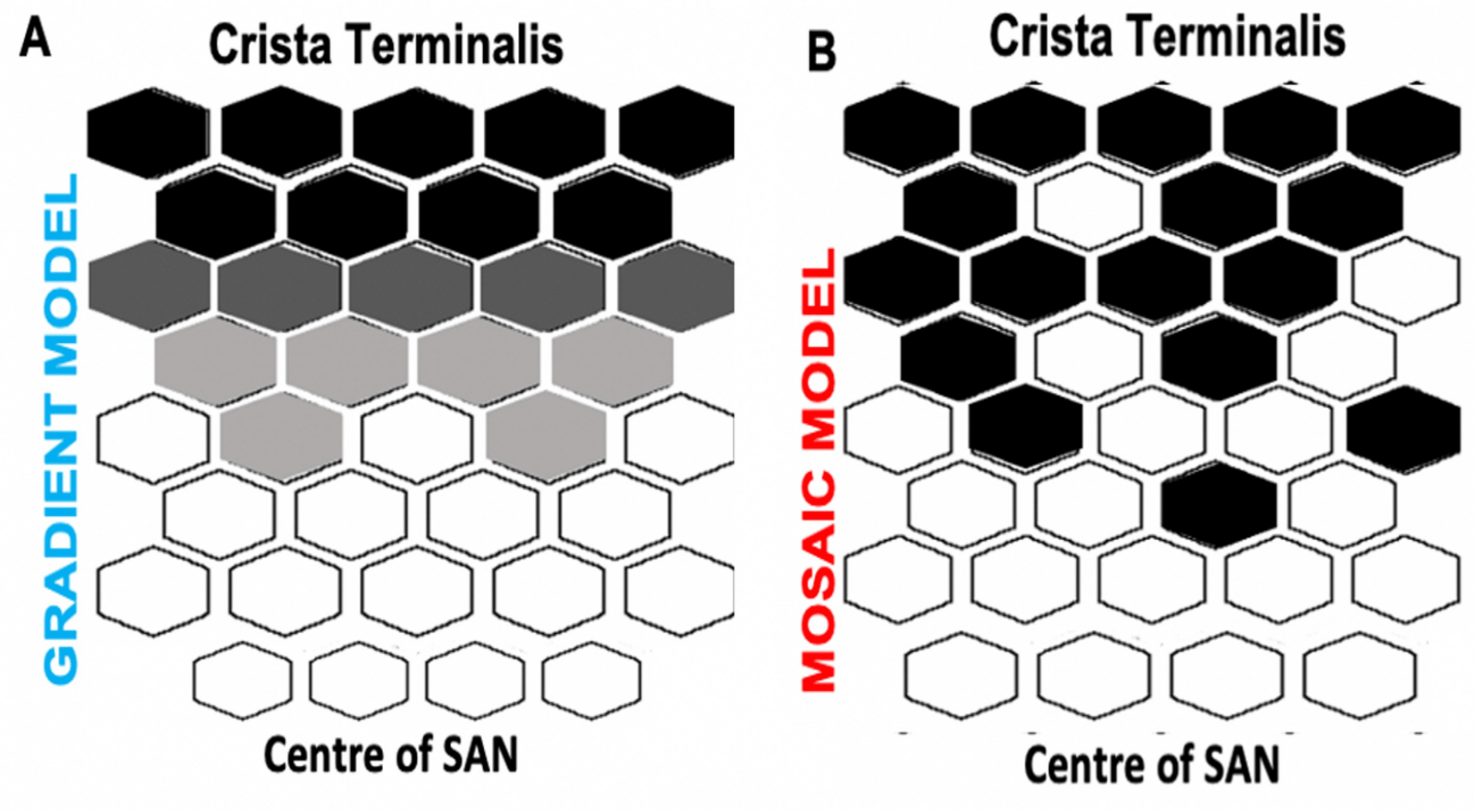
A diagram comparing the two suggested models of cell arrangement in the SAN The white hexagons represent the sinoatrial node (SAN) cells, black hexagons represent atrial cells. (A) The “gradient” model shows a steady change from SAN cells to transitional cells (grey) from the centre of SAN to the periphery. (B) Model B appears like a “mosaic”, with a progressively declining SAN cell: atrial cell fraction from the centre of SAN to the periphery.

There have been two suggested interpretations of the cellular arrangement within the SAN. In one model, it is proposed that there is a gradual change between the cells located in the centre of the SAN and those situated closer to the crista terminalis, whereby the peripheral cells are larger, possess an inherently faster pacemaking rate, and have the transitional qualities of both the atrial and pacemaker cells. This is known as the “gradient” model [24] (Figure 2A). On the other hand, Verheijck and colleagues have proposed the “mosaic” model, which suggests that pacemaker cells are evenly distributed within the SAN, regardless of differences in automatic pacemaking rate [26]. This model also indicates the presence of atrial cells in the nodal region, with the peripheral regions of the SAN closest to the crista terminalis containing a higher population than the SAN centre [26,27] (Figure 2B).

Paranodal Region

A distinct paranodal area surrounds the SAN (Figure 1) [28]. Although the function of this area is not entirely established, it is considered to act as an auxiliary pacemaker that has the potential to become the leading pacemaker in the event of SAN disease [28]. The paranodal region is regarded as transitional tissue, serving as an intermediary between the SAN pacemaker cells and the atrial myocytes [28]. Even though the paranodal cells display characteristics similar to those of the nodal region, the specific ion channels located in this area are observed to differ from those in the neighbouring SAN and atrial tissues [29]. This is thought to result from the paranodal expression of ion channels, namely the sodium and inward-rectifier potassium ion channels, which are intermediate between these tissues [25,29]. It should be noted that within this region, some of the paranodal cells exhibit markers specific to the atria, such as connexin-43 (a gap junction protein) and the atrial-specific natriuretic peptide (ANP), whereas other paranodal cells exhibit a cellular architecture closely resembling that of the SAN [25,29]. As the paranodal area shows cellular regions with a phenotype closely resembling that of the SAN, it has been proposed that this region could be a novel target in the development of bio-pacemakers [28] using cell transplantation and gene transfer methods to mimic the pacemaking action of the SAN [30]. This therapy has been suggested as a potential treatment for pacemaker dysfunction seen in conditions such as sick sinus syndrome, while also avoiding the limitations and risks associated with current electronic pacemaker treatments, such as the risk of infection during pacemaker implantation [31].

Imaging of the SAN

The Limitations of Serial Histological Sectioning Techniques 

Our contemporary knowledge of the human SAN and the supporting electrical conduction structures of the heart is the result of meticulous reconstruction of two-dimensional serial histological sections [11,32-35]. However, one of the key limitations of this approach is the restricted three-dimensional resolution caused by the gaps between sequential sections, a distance usually measuring 60-340 µm [33,34]. Moreover, such examination is constrained by the need to use samples from isolated portions of the heart, which are small-scale, require frequent sectioning, and are subject to the often destructive methods of serial sectioning techniques [36]. Thus, contextualising the individually examined samples within the setting of the entire cardiac conduction system is challenging and susceptible to inaccuracies, making it difficult to represent the conduction system in its precise cardiac positions [36]. Stephenson et al. (2017) indicated that such procedures do not always accurately depict the three-dimensional complexity of the heart’s structures, leading to varied and often erroneous reports of both the arrangement of non-pacemaker tissue and the organisation of the conduction system [37].

Nevertheless, extensive studies have shown that despite these limitations, traditional methods that rely on immunohistochemical and histological analysis continue to be vital techniques that corroborate contemporary three-dimensional imaging methods [37,38].

The Micro-computed Tomography Approach

Micro-computed tomography technology is regarded as a revolutionary method for mapping the electrical conduction system of the heart and is considered to provide superior visualisation of the three-dimensional structure of the human SAN and the morphology of the cardiac conduction system [39]. In their detailed study of the three-dimensional microanatomy of the human heart’s conductive structures, Stephenson et al. (2011) demonstrated that this technique overcomes several challenges associated with traditional methods [39]. For example, it preserves the three-dimensional organisation of cardiac structures, as this method is non-destructive to the sampled tissue. There is no alteration of the tissue resulting from the dissecting and freezing procedures required in traditional methods such as serial sectioning [39]. Moreover, this technique is faster than traditional methods, with the study reporting a processing time of 20-50 minutes [39], whereas MRI procedures can take several hours [39], and equivalent serial section methods may require several days. Additionally, compared to traditional sectioning techniques, the micro-CT approach provides greater resolution, allowing for a more precise representation of the conduction system and clearer distinction of the SAN from the surrounding atrial structures [39]. Furthermore, the ability to reverse the staining procedure allows the tissue samples to be preserved for future use and analysis [39].

Therefore, using such innovative methods to produce high-quality three-dimensional images of the human cardiac conduction structures may enhance our understanding of the morphological differences between normal and diseased hearts, as well as better identify age-related changes in specific structures such as the SAN [39]. These methods may also complement the instrumental work of Chandler et al. (2011), who produced a 3D anatomical model of the human SAN using traditional immunohistochemical, histological, and MRI techniques [11]. Thus, newer imaging methods such as micro-CT may support the development of anatomically and physiologically accurate models of the human heart, contributing to the goal of creating a “virtual heart” [39].

Function of the SAN 

Location of SAN Impulse Initiation

As discussed in the preceding sections, the SAN is the heart’s principal pacemaker and the site of cardiac impulse initiation [4]. Originating typically in the central regions of the SAN complex (Figure 1), which contains the self-excitable pacemaking cells, the action potential is then propagated to the peripheral regions of the SAN and spreads into the surrounding muscle of the crista terminalis [40]. However, it must be noted that in humans, heterogeneity has been observed regarding the site of primary pacing within the SAN. The primary site is therefore widely considered to be the region where the generation of a spontaneous action potential is the quickest [41]. Furthermore, it has been well established that the location of the primary pacemaker cells within the SAN can shift according to certain factors, such as autonomic influence [42]. In addition, it has been proposed that there is a grading of pacemakers within the SAN complex, such that the higher the site, the quicker the heart rate [42]. Under sympathetic nervous drive, for instance, there is an upward shift of the primary site, resulting in an increase in the heart rate [42]. Boyett et al. (2000) considered the shift of the pacemaker to be the result of differences in ion channel expression in the central SAN cells compared with the peripheral SAN cells [25]. Correspondingly, a shift in the pacemaker position may be induced by specific ion channel inhibitors, which move the site to regions where automaticity is less dependent on those respective channels [25]. This theory has been substantiated through numerous experiments using f-channel [43], Ca^2+^ [44], and K^+^ [45] channel blockers.

Spontaneous Mechanisms of the SAN

The pacemaker function of the SAN has been extensively researched following its discovery in 1907 [3]. The initial interpretations of the diastolic depolarisation of pacemaker cells were described by Brown et al. (1982) [46] and later by Irisawa et al. (1993) [47] in relation to the stimulation of specific ion currents. The SAN pacemaking function was initially thought to result from a decay in outflowing K^+^ currents (Ik2) during depolarisation at diastole [48]. However, this notion was reconsidered in the 1970s following DiFrancesco and colleagues’ identification of the so-called “funny” channels and their corresponding “funny” current (I_f_) in the SAN [49]. These channels were termed “funny” because of their unusual properties-they are permeable to both Na^+^ and K^+^ ions, are activated upon hyperpolarisation, and do not exhibit fast kinetics [50]. There are four different isotypes of the hyperpolarisation-activated cyclic nucleotide-gated channels (HCN1-4); however, the key pacemaker channel expressed in the human SAN is the HCN4 channel, which generates I_f_ [30,51].

I_f_ is initiated upon membrane hyperpolarisation between values of approximately −40 mV and −45 mV, resulting in membrane depolarisation [51]. The identification of an overlapping range in which SAN diastolic depolarisation (about −40 mV to −65 mV) arises (Figure 4A) and the voltage at which I_f_ is initiated reinforced the concept that this current could serve as a suitable mechanism for initiating the diastolic stage, functioning as a so-called “pacemaking current” [51].

**Figure 3:**
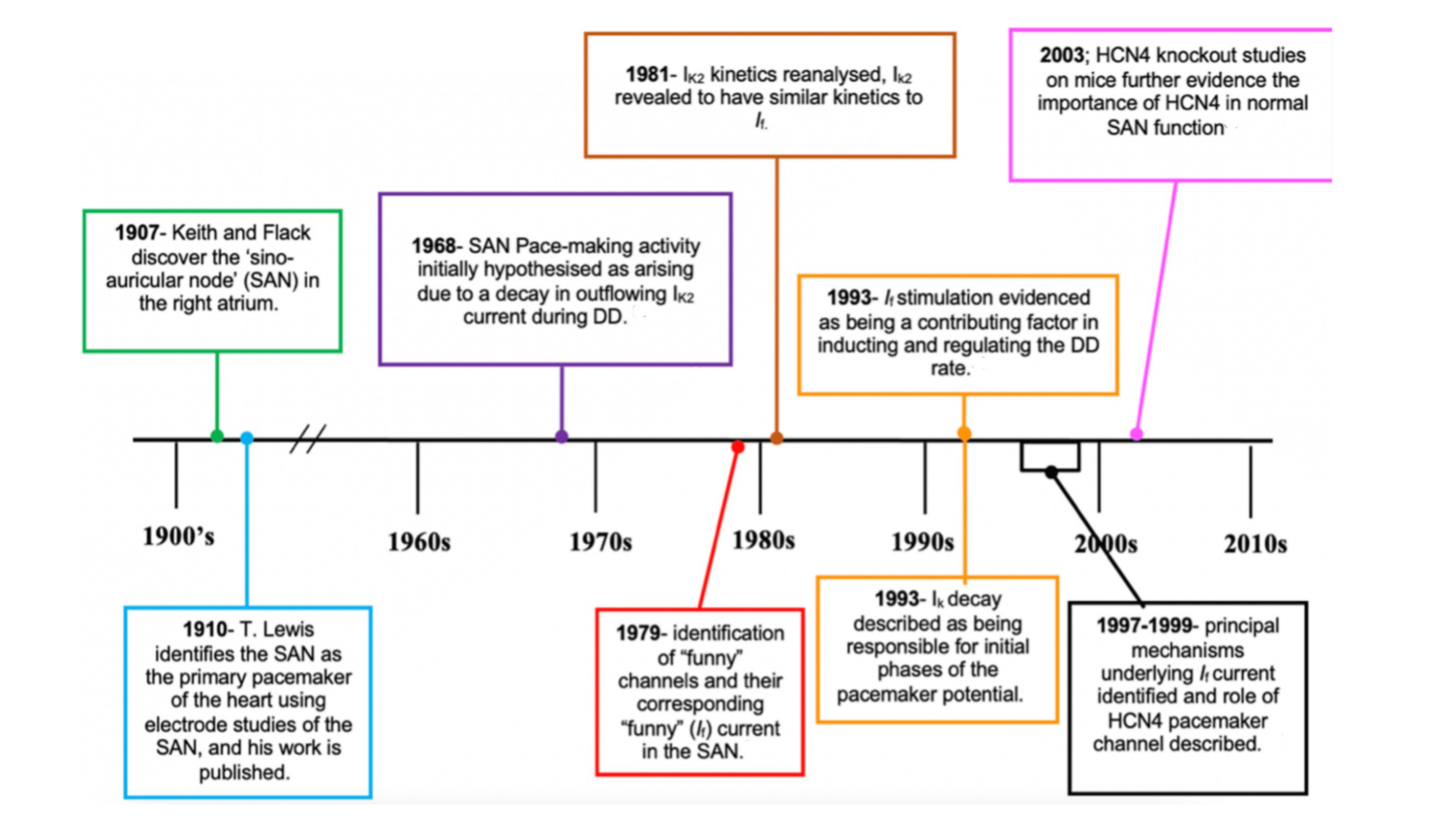
A summary of the historical progress in our understanding of diastolic depolarisation (DD) and the role of the funny current (If) in the pacemaker potential that have been discussed in this review The author made this figure with the information adapted from Mangoni et al. (2008) [53].

**Figure 4:**
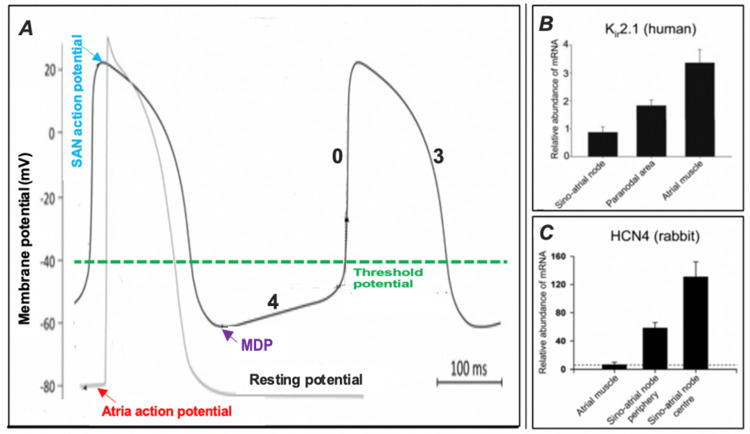
SAN pacemaker potential (A) Action potentials of the SAN (marked by blue arrow) and atria (marked by red arrow). Phase 4: Initial decay of IK, followed by a surge in I_f_ facilitated by HCN4 pacemaker channels, leading to amplified Na^+^ and K^+^ inward conductance; opening of the T(transient)-type Ca^2+^ channels, thus increasing the Ca^2+^ current into the cell; diastolic depolarisation. Phase 0: Activation of longer-lasting L-type Ca^2+^ channels, hence a corresponding amplification of inward Ca^2+^ conductance, depolarising the SAN pacemaker membrane. Slow inward Ca^2+^ movement and absence of greater Na^+^ currents; therefore, the upstroke gradient of this phase is lower in the SAN compared to the atrial muscle. Phase 3: Opening of K^+^ channels and resultant surge in K^+^ outward conductance leading to SAN cell membrane repolarisation. Phases 1 and 2: Absent in SAN pacemaker cells. (B) Healthy human hearts’ mRNA levels of Kir2.1 expression are lowest in the SAN. (C) mRNA levels of HCN4 (data obtained from the heart of a rabbit); expression is highest in the centre of the SAN.

This concept has been further supported by the identification of higher HCN4 expression in the SAN and its absence in the atrial muscle (Figure 4C), validating the significance of I_f_ in generating SAN pacemaker activity [52], while HCN4 mutations have been associated with SAN dysfunction (Figure 3) [53]. Nevertheless, this discovery does not invalidate the role of Ik decay in the initial stages of the pacemaking potential [47].

This finding contributes to a more contemporary understanding of pacemaker activity, specifically highlighting the role of inward I_f_ current activation during diastolic depolarisation as a key driver of automaticity [54]. The I_f_ current has been comprehensively studied, and its role in SAN pacemaker function has been widely examined over the years [55-57]. Numerous studies conducted after its discovery, including more recent reviews [58-60] and experimental analyses of I_f_ function in the human SAN, have provided further convincing evidence that I_f_ plays a central part in the so-called “membrane clock”, which enables the SAN to regulate the heart rate [61]. However, this concept continues to be extensively investigated in light of the growing recognition of complex cellular mechanisms contributing to all aspects of impulse initiation and maintenance [62]. Among these is the identification of a “Ca^2+^ clock”, in which spontaneous pulsatile discharge of Ca^2+^ by the smooth endoplasmic reticulum is also thought to contribute to SAN pacemaking activity [62]. 

Another main influence on SAN pacemaking is the lower expression of the channel Kir2 (specifically the Kir2.1 isoform) in the SAN (Figure 4B), which is an inward rectifying K^+^ channel [52]. As a result, the SAN has no corresponding IK1 current [52]. Therefore, both the absence of IK1 and the presence of I_f_ result in the SAN not exhibiting a steady “resting” potential, and both are contributing factors to SAN pacemaking activity [52]. This is supported by Miake et al. (2002), who demonstrated that Kir2.1 silencing in the ventricular muscle can lead to pacemaker activity developing in this originally non-pacemaker region [63]. As such, in the periods between actual excitation potentials, the SAN membrane potential demonstrates what is referred to as a phase 4 depolarisation, a spontaneous depolarisation occurring during cardiac diastole that is slower in nature, known as the “pacemaker potential” [44] (Figure 4A). This has been a well-established concept explaining the mechanism by which the SAN displays automaticity and has been described as early as the 1940s [64].

Consequently, the SAN pacemaker demonstrates a “maximum diastolic potential” (MDP), which demarcates the lowest membrane potential achieved by the cells between successive periods of depolarisation or action potentials (Figure 4A) [65]. Therefore, this decreased IK1 (which is in fact an outflowing K^+^ current) and I_f_ initiation cause the SAN pacemaker cells to approximate an MDP value of about −55 mV, whereas a value of about −70 mV is apparent in the paranodal cells. In comparison, the resting potential of the atrial cells is in the region of −85 mV [66]. From this MDP, the cells of the SAN reach a membrane potential of −40 mV, the minimum potential required to trigger an action potential, as a result of this already present pacemaker potential [63-65]. Consequently, the pacemaker cells of the SAN demonstrate spontaneous action, and the gradient of phase 4 depolarisation, which is unique to these cells, is a critical element governing the rate of impulse generation and, therefore, sinus rhythm and heart rate [67-69].

Regulating SAN automaticity

Automaticity denotes the capability of specialised cells in the heart to demonstrate spontaneous depolarisation and discharge of recurrent action potentials [70]. The intrinsic firing rate of the SAN is around 100-110 action potentials per minute (Figure 4A). Extrinsic and intrinsic regulation lowers this to an average of 60-80 or 100 bpm [70]. It is the SAN, in healthy individuals, that establishes the principal intrinsic rate [4]; therefore, other structures such as the Purkinje fibres and the atrioventricular node, which also exhibit automaticity, are deemed secondary pacemakers, as they take over action potential initiation only in the event of unsuccessful SAN impulse initiation or propagation [70]. Additionally, the distinct paranodal region may take over the function of the SAN in cases of SAN disease [70], as discussed in the "Paranodal Region" section.

Intrinsic SAN Regulation: microRNA Control of HCN4 Expression

Initial efforts to comprehend the principal mechanisms underpinning the SAN pacemaking abilities were made during the 1970s [71] (Figure 3), and such efforts are still ongoing. Nearly all cells in the heart that demonstrate spontaneous activity show I_f_ channel expression [72]. DiFrancesco and colleagues have shown I_f_ channels, primarily the HCN4 channels, to be a main ionic influence underpinning SAN automaticity and pacemaking activity [73]. In 2020, Petkova and colleagues identified vital non-coding microRNAs that regulate the pacemaker function of the human SAN [74]. This ground-breaking study confirmed that there is indeed a unique presentation of microRNAs in the SAN compared to the atria, and this is thought to influence the expression of key molecules responsible for the pacemaking function of the human SAN, such as HCN4 expression [75]. In their analysis, the team demonstrated, in particular, that the microRNA miR-486-3p governs the expression of the HCN4 mRNA (and therefore the HCN4 protein) and established this notion through dedicated gene assays [76]. They then showed that when miR-486-3p is robustly expressed in the SAN, it can downregulate the mRNA of HCN4 and the subsequent HCN4 protein concentrations, thus reducing the SAN action potential rate [76]. This supported earlier studies in athletically trained mouse models, where it had been shown that diminished expression of HCN4 and the subsequent sinus bradycardia after training could be attributed to miR-486-3p SAN overexpression [77].

In conclusion, they showed miR-486-3p and other microRNAs to be more pronounced in the musculature of the atrium than in the SAN, and it is these microRNAs that function to inhibit HCN4 and other key pacemaking molecules, respectively [76]. Therefore, it has been proposed that if the expected activity of these microRNAs is as such, it could further elucidate why atrial cardiomyocytes do not physiologically exhibit pacemaking capabilities, while the reduced expression of miR-486-3p and other microRNAs in the node can further explain SAN pacemaker abilities [76].

A novel treatment for sinus tachycardia: a possible look into the future?

The role of microRNAs in cardiac function remains an active area of investigation, with contemporary research increasingly focusing on their regulatory influence over cardiac pacemaker activity and their potential applications in the diagnosis and therapeutic management of cardiac dysfunction [78,79]. For example, the inhibition of HCN4 mRNA and the subsequent decrease in HCN4 channel expression by miR-486-3p may provide a novel therapy for treating sinus tachycardia (a condition where the heart rate is elevated above normal) and may act as a suitable replacement for currently used pharmacological interventions such as Ivabradine [76-79]. Unlike miR-486-3p, Ivabradine does not inhibit HCN4 at the transcriptional level; it works by the selective direct blockage of the HCN4 channel [80]. Therefore, as blockage of the HCN4 channel interrupts ionic movement during I_f_, the rate of diastolic depolarisation becomes lengthened, hence elongating the duration of phase 4 (Figure 4A). As a result, the SAN pacemaking rate becomes lowered, leading to a subsequent decrease in heart rate [81,82]. Moreover, Ivabradine’s effects on the heart are exclusive to the SAN; therefore, it is thought not to have an influence on the cardiac contractile force or repolarisation of the ventricles [83]. Perhaps miR-486-3p may also show similar exclusivity to the SAN, though this remains to be elucidated. The effectiveness of Ivabradine is dependent on the given dose; therefore, quantities have to be modified in accordance with the heart rate at rest and how well the patient tolerates the drug in order to attain a lower heart rate, ideally in the range of 50-60 bpm at rest [84].

A major contraindication of Ivabradine, however, is the use of the drug during pregnancy [84]. Ivabradine use during the embryonic organogenesis window has been linked to foetal bradycardia and a resulting hypoxic status, causing possible developmental abnormalities and, more extremely, foetal death [85]. Hence, in consideration of the foetal risk factors, it is strongly suggested that women who are preparing for pregnancy or are currently pregnant (in the setting of tachycardia) should be prescribed an alternative drug with a lower threat to the unborn, such as beta-blockers [85]. However, it has been suggested that it may be possible to reduce the risk of foetal toxicity in tachycardic pregnant women or women preparing for pregnancy by using a novel therapy such as the aforementioned non-coding miR-486-3p, although as of yet, this has not been evidenced through dedicated clinical trials. What is evident, though, is that remedial use of microRNAs in clinics is a matter that is being extensively researched, with a number of microRNAs making it to phase 1 and phase 2 of clinical trials [86]. An example of this is miR-16, which currently has a phase 1 clinical status. It is thought that if this microRNA performs as expected, it could be a ground-breaking therapy for mesothelioma, an aggressive form of lung cancer [86]. Therefore, utilisation of miR-486-3p’s ability to up- or down-regulate HCN4 protein expression in the SAN may indeed offer a newer and possibly less risky treatment for sinus tachycardia [76].

## Conclusions

To conclude, this review has summarised key aspects of SAN anatomy and its role in maintaining the heart’s rhythm. The understanding of SAN automaticity has evolved through continued investigation of the mechanisms that drive pacemaker activity, including the cellular and molecular processes that sustain rhythmic firing. Advances in imaging and modelling techniques continue to enhance insight into SAN structure and function, offering potential pathways for developing accurate computational models of cardiac rhythm. In addition, molecular regulators such as microRNAs are emerging as important factors in modulating pacemaker function and may one day provide alternative strategies for managing tachycardia. Although further study is needed, these developments highlight the growing potential of molecular and technological approaches to improve understanding and treatment of rhythm disorders.
